# C-855-06. Evaluation of Accuracy and Clarity of AI Burn First Aid Responses

**DOI:** 10.1093/jbcr/irag033.090

**Published:** 2026-04-06

**Authors:** Michelle Ahn, Philip H Chang

**Affiliations:** Columbia University, NY, New York; William Randolph Hearst Burn Center, NY, New York

## Abstract

**Introduction:**

AI platforms have surged to the forefront of information acquisition with burn patients turning to AI instead of classic search engines for initial treatment of burn injuries. However, concerns have been expressed about the quality of information that current AI models utilize and AI hallucinations. Our team sought to evaluate accuracy and clarity of AI responses to inquiries regarding common burn injuries.

**Methods:**

5 scenarios pertaining to burns of common etiologies were formulated, each with 2 queries differentiating pediatric and adult injuries. These queries were then submitted to the most commonly accessed AI platforms available to the US public. AI responses were then reviewed by burn professionals who rated clarity and accuracy using a Likert scale from 1-10, with 1 being least accurate and clear to 10 being completely accurate and understandable. Each search engine was opened in incognito mode and refreshed before every submission.

**Results:**

Overall, information generated by the queries was reasonably accurate with some variation in specific details as accuracy scores ranged from 7.4 to 8. Clarity of the responses was judged very good with scores from 7.9 to 8.1. Differences in reply tone to pediatric queries were observed with more sympathy than adult queries. Variable accuracy in information was noted regarding time length for cool running water to burn injuries as well as use of plastic wrap to dress the wound. Queries regarding electrical burn injury and radiator injury generated preventive suggestions in addition to initial first aid.

**Conclusions:**

Previous analyses of online information accuracy accessible through common search engines raised significant concerns about the quality of clinical recommendations available. The AI generated responses were deemed reasonably safe. Despite stylistic differences in the responses generated, clarity scores do not reflect variability in the tone of the responses. As AI algorithms evolve, the burn community should continue to monitor the accuracy and clarity of AI generated responses to ensure safe initial responses to injuries are appropriately communicated.

**Applicability of Research to Practice:**

Burn teams should be aware of the quality and variety of online information accessible to patients with regards to initial recommended treatment which affect clinical prognosis. The burn community directly impacts the quality of AI generated inquiries by providing information on individual center websites and through peer reviewed literature.

**Funding for the study:**

N/A.

